# The role of dietary supplements, including biotics, glutamine, polyunsaturated fatty acids and polyphenols, in reducing gastrointestinal side effects in patients undergoing pelvic radiotherapy: A systematic review and meta-analysis

**DOI:** 10.1016/j.ctro.2021.04.006

**Published:** 2021-04-23

**Authors:** Benjamin Bartsch, Chee Kin Then, Elinor Harriss, Christiana Kartsonaki, Anne E. Kiltie

**Affiliations:** a Oxford Institute for Radiation Oncology, Department of Oncology, University of Oxford, Oxford, UK; b Bodleian Health Care Libraries, University of Oxford, Oxford, UK; c Clinical Trial Service Unit & Epidemiological Studies Unit (CTSU), Nuffield Department of Population Health, University of Oxford, Oxford, UK; d Medical Research Council Population Health Research Unit (MRC PHRU) at the University of Oxford, Nuffield Department of Population Health, University of Oxford, Oxford, UK

**Keywords:** Dietary supplements, Meta-analysis, Pelvic radiotherapy, Biotics, Gastrointestinal toxicity, Systematic review

## Abstract

**Background and purpose:**

Pelvic radiotherapy (RT) often results in gastrointestinal toxicity and clinical trials have demonstrated a potential benefit of dietary supplements in alleviating acute effects. However, no prophylactic agents have been approved to date for relief of gastrointestinal side-effects caused by pelvic radiation. In this systematic review, we evaluated the efficacy of dietary supplements in preventing or alleviating symptoms of gastrointestinal toxicity in patients undergoing pelvic RT.

**Materials and methods:**

CENTRAL, MEDLINE, EMBASE, and ClinicalTrials.gov were searched up to June 2020 for randomised controlled trials. Interventions included four supplement categories: biotics, glutamine, poly-unsaturated fatty acids and polyphenols. Efficacy was determined with reference to outcomes based on symptoms of acute gastrointestinal toxicity, including diarrhoea, nausea, vomiting, flatulence/bloating, bowel movement frequency, tenesmus and rectal bleeding.

**Results:**

Twenty-three randomised controlled trials (1919 patients) were identified in this review. Compared with placebo, probiotics (RR = 0.71; 95% CI: 0.52 to 0.99), synbiotics (RR = 0.45; 95% CI: 0.28 to 0.73) and polyphenols (RR = 0.30; 95% CI: 0.13 to 0.70) were significantly associated with a lower risk of diarrhoea. Biotic supplements also reduced the risk of moderate to severe diarrhoea (RR = 0.49; 95% CI: 0.36 to 0.67) and the need for anti-diarrhoeal medication (RR = 0.64; 95%CI: 0.44 to 0.92). In contrast, glutamine had no effect on acute symptoms (RR = 1.05; 95% CI: 0.86 to 1.29). There was a non-significant trend for reduction in nausea and mean bowel movements per day using dietary supplements.

**Conclusions:**

Biotic supplements, especially probiotics and synbiotics, reduce acute symptoms of gastrointestinal toxicity in patients undergoing pelvic radiotherapy.

## Highlights

• Probiotics and synbiotics reduced the risk of diarrhoea after pelvic radiotherapy.
• Polyphenols, but not glutamine, may reduce the risk of diarrhoea.
• Biotic supplements reduced the need for anti-diarrhoeal medication.
• Large clinical trials are needed of biotics with modern radiotherapy techniques.

## 1 Introduction

Radiotherapy is a major cancer treatment modality, used to treat approximately 50% of patients [1]. Over 200,000 patients in the US are treated with pelvic or abdominal radiotherapy each year [2]. It is inevitable that normal gastrointestinal tissues are exposed to radiation during pelvic radiotherapy [3], with approximately 80% of patients developing acute symptoms of radiation-induced gastrointestinal toxicity [4]. However, despite their impact on patients’ quality of life, no prophylactic agents for the alleviation of gastrointestinal side-effects from pelvic radiation have been approved to date [5].

Acute symptoms usually develop during or immediately after RT, and typically improve within three months following RT [6]. The most common acute side effect is diarrhoea, affecting up to 80% of all patients [7]. Other symptoms, such as abnormal stool output, vomiting, rectal bleeding, tenesmus and gastrointestinal discomfort are also common. Late symptoms include GI bleeding, fistula, stricture and colostomy [8].

Use of a dietary supplement is aimed at boosting daily intake of specific nutrients, to much higher levels than obtained from the diet, to alleviate symptoms of gastrointestinal toxicity. Such dietary supplements include biotics, glutamine, poly-unsaturated fatty acids (PUFAs) and polyphenols. Probiotics, mainly of the *Lactobacillus* and *Bifidobacteria* genera, are live microorganisms thought to produce health benefits following passage to the intestine [9]. Prebiotics are soluble or non-soluble dietary fibres, that pass undigested through the upper gastrointestinal tract and are metabolised by bacteria in the colon, thus altering gut microbiota beneficial to the host’s health [10]. The use of synbiotics refers to administration of a combination of prebiotics and probiotics; the presence of the prebiotic enhances survival of the probiotics in the lower gastrointestinal tract. Administration of biotics can enhance production of key metabolites, particularly SCFAs, and butyrate reduces mucosal inflammation and promotes epithelial repair following injury [11]. Glutamine, poly-unsaturated acids (PUFAs) and polyphenolic compounds have also been employed in supplement intervention strategies in pelvic RT. Anti-inflammatory effects of the omega-6 PUFA conjugated linolenic acids are seen in inflammatory bowel disease [12]. Glutamine is the most abundant amino acid with important roles in support of mucosal growth and function. It can protect the oral and intestinal mucosa from radiation damage by improving nitrogen balance and detoxifying normal host tissue [13], [14], [15]. Polyphenolic compounds extracted from plants protect tissues against oxidative stress from ROS and RNS, both of which are products of radiotherapy [16].

This review tests the hypothesis that administration of oral dietary supplements for cancer patients receiving pelvic radiotherapy may trigger changes in the lower gastrointestinal tract which lead to a reduction in gastrointestinal toxicity.

## 2 Material and methods

### 2.1 Trial registration number

The study protocol was published on the PROSPERO international prospective register of systematic reviews (registration number CRD42020183304).

### 2.2 Search strategy and study selection

The following electronic databases were searched from inception to the search date (19/06/2020) for relevant literature: Cochrane CENTRAL, Ovid Medline, Ovid Embase, and ClinicalTrials.gov. The search strategies included both medical subject heading and free text terms to retrieve relevant RCTs and non-randomised studies regarding gastrointestinal side effects in cancer patients undergoing pelvic radiotherapy, limited to studies in humans only. The full set of search strategies is available in Appendix A to C, and protocol details are available in the PROSPERO registration [17]. Relevant articles were identified on PubMed. Handsearching of meta-analyses, systematic reviews and papers identified studies not indexed in the electronic databases used for this review. All titles and abstracts retrieved by electronic searches were downloaded and duplications removed using EndNote reference management software.

### 2.3 Data extraction

Systematic data collection from included studies was conducted using a data collection form designed specifically for this review. It included the following information (where available) for each dataset: publication year, study design, participants (number, age distribution, gender distribution, details of malignancy, details relevant to inclusion and exclusion criteria), current cancer treatment, intervention and measured outcomes.

### 2.4 Outcome assessment

Different measures of treatment effects were used for dichotomous and continuous outcomes, namely, risk ratio (RR) for dichotomous outcomes and the mean difference between the intervention and control arms for continuous outcomes. Standardised mean difference was used to compare results from studies that reported the same outcomes measured on different scales.

### 2.5 Study quality, assessment of heterogeneity, publication bias and quality assessment

Risk of bias assessment was carried out for all studies that met the inclusion criteria, using the Cochrane Risk of Bias 2 tool. To assess the heterogeneity, we used a chi-squared test and *I*^2^. P values less than 0.1 were considered as evidence of heterogeneity. Tau-squared is the estimated standard deviation of underlying effects across studies. Begg’s funnel plots were used to visually assess asymmetry potentially due to publication bias. Quality assessment was conducted using GRADEpro online software [18].

### 2.6 Data synthesis and statistical analysis

Meta-analyses were performed to measure the effect of dietary supplements on an outcome, in instances where there were three or more studies that reported the same outcome. All analyses were conducted using RevMan 5.4 and R version 4.0.2 with package ‘meta’. For dichotomous outcomes, RR were estimated and were meta-analysed using a random effects model using the Mantel-Haenszel method. For continuous outcomes, mean differences were estimated and were pooled using a random effects model with the inverse variance method. 95% confidence intervals (CI) for all estimates were calculated. Meta-regression was used to assess whether the effects on incidence of diarrhoea varied by study characteristics.

## 3 Results

**Fig. 1:**
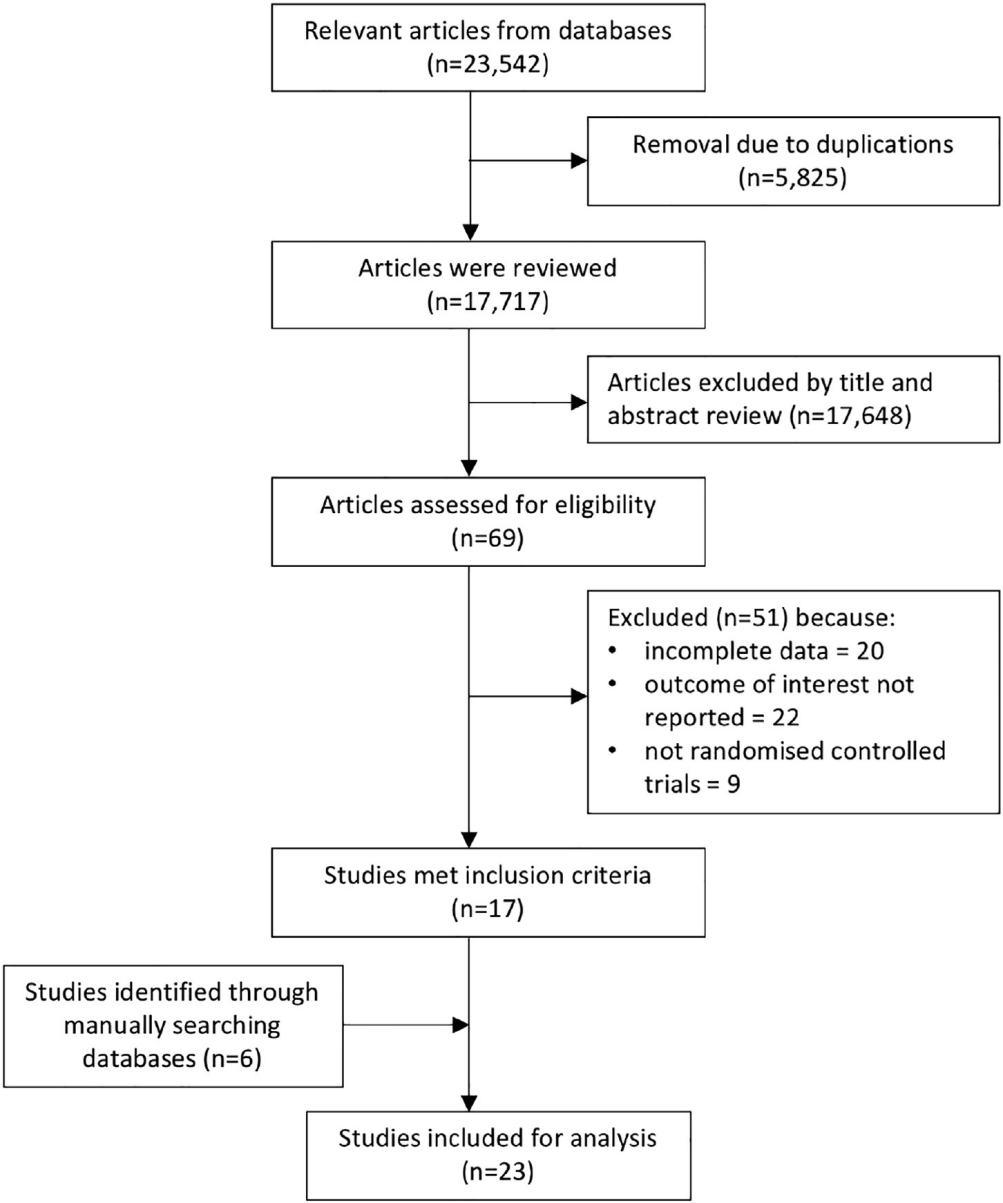
PRISMA flow chart of studies evaluated in the systematic review.

**Fig. 2:**
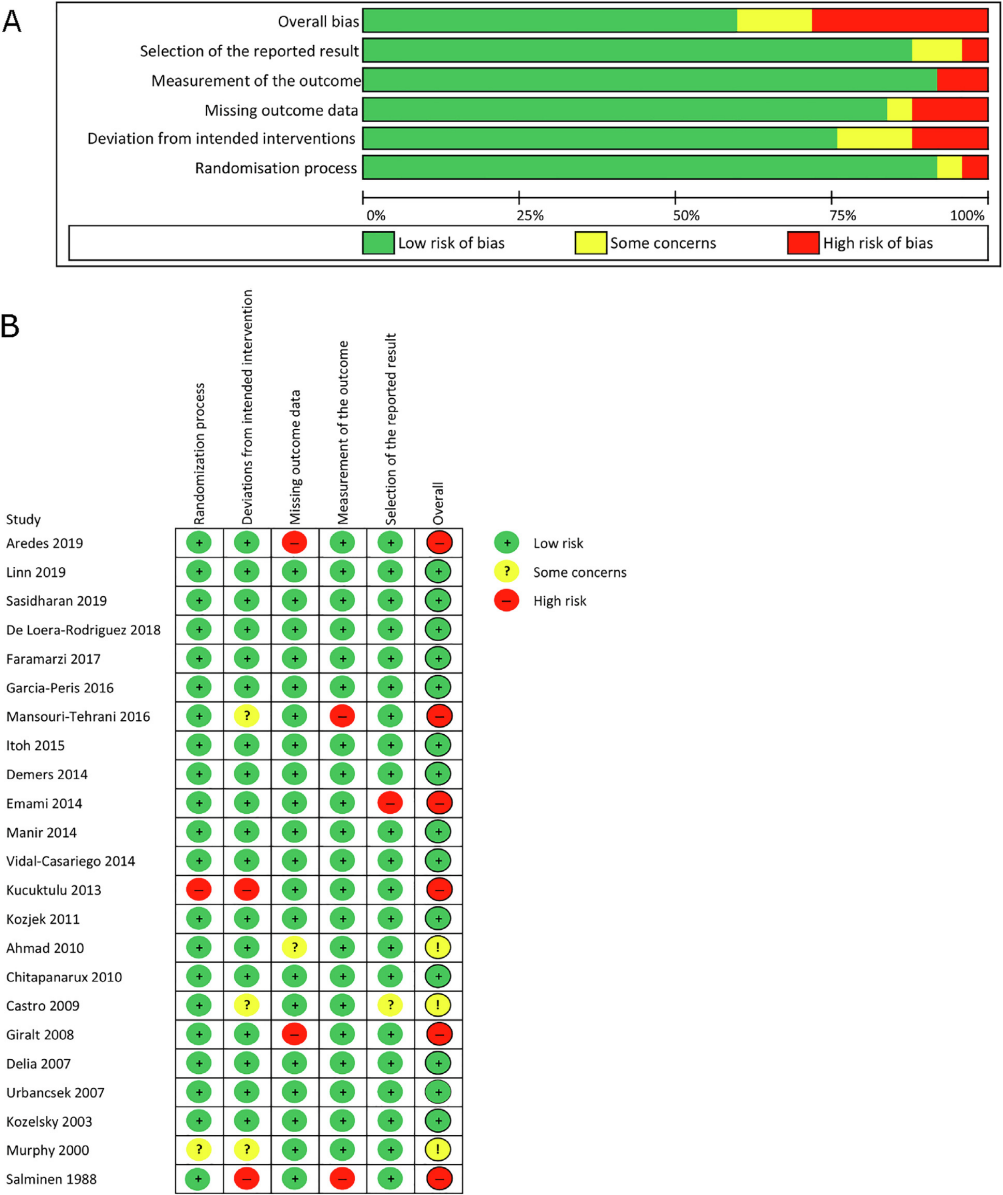
Risk of bias summary for all studies that met the inclusion criteria.

The search of the four primary databases identified 23,542 titles published between 1946 and June 2020 (search process summarised in Fig. 1). After 5825 duplications were removed, a total of 17,717 entries remained. These studies were manually reviewed by title and abstract and 17 met the inclusion criteria. Six further studies were identified from manual searches of the reference sections of research articles. Finally, 23 studies met the inclusion criteria and could be used for quantitative analysis. There was no evidence that the effects of interventions on incidence of diarrhoea varied by mean age (p = 0.552), proportion of male participants (p = 0.131), sample size (p = 0.131) or RT dose (p = 0.073) (Figure S1). Results of the overall and individual risk of bias assessments for each of the five domains are presented in Fig. 2.

### 3.1 Included studies and characteristics of included studies

In total 23 studies involving a total of 1919 patients met the inclusion criteria and for each outcome, they were grouped by intervention category. These studies were all randomised controlled trials and their characteristics are shown in the Table 1, Tables S1 and S2. In total, the trials included in the review reported ten different relevant symptoms, as shown in Table S3.

**Table 1:** Characteristics of included studies.

Study	Cancer type	Intervention	Sample size	Mean age (years)
**Biotics**				
Sasidharan, 2019 [47]	Cervical	Prebiotic: resistant starch	100	48.0
Linn, 2019 [48]	Cervical	Synbiotic: *L. acidophilus, B. animalis* andyoghurt	54	54.8
De Lorea-Rodriguez, 2018 [49]	Cervical	Synbiotic: *L. acidophilus, B. lactis* and inulin	70	49.9
Mansouri-Tehrani, 2016 [50]	Colorectal, prostate, endometrial, bladder, ovary, cervix, bone sarcoma	Synbiotic: *S. thermophiles, Lactobacilli, Bifidobacter* and honey	46	62.0
Garcia-Peris, 2016 [51]	Gynaecological - cervical, endometrial, vulval-vaginal, uterus	Prebiotic: inulin oligosaccharide and fructo-oligosaccharide	38	60.3
Itoh, 2015 [52]	Cervical	Prebiotic: hydrolysed rice bran	20	49.3
Demers, 2014 [53]	Gynaecological, rectal or prostate	Probiotic: *L. acidophilus* and *B. longum*	148	61.2
Chitapanarux, 2010 [54]	Cervical	Probiotic: *L. acidphilus* and *B. bifidum*	63	47–52*
Castro, 2010 [35]	Cervical or endometrial	Probiotic: *L. casei* and *B. breve*	40	–
Giralt, 2008 [19]	Cervical or endometrial	Probiotic: *L. casei*, *S. thermophiles* and *L. delbrueckii*	85	60.1
Delia, 2007 [33]	Cervical or rectal	Probiotic: Four strains of *lactobacilli*, three strains of *bifidobacteria* and one strain of *streptococcu*s	482	–
Urbancsek, 2001 [55]	Uterus, ovarian, prostate, rectal	Probiotic: *L. rhamnosus*	205	59.5
Murphy, 2000 [22]	Prostate, gynaecological	Prebiotic: psyllium	60	64.5
Salminen, 1988 [56]	Cervical, uterus	Synbiotics: *L. acidophilus* and lactulose	21	40–75

**Glutamine**				
Vidal-Casariego, 2014 [20]	Prostate, bladder, cervical, endometrium, rectal	Glutamine	65	66.5
Manir, 2014 [57]	Cervical, rectal, endometrium, prostate	Glutamine	85	56.7
Kucuktulu, 2012 [58]	Rectal, bladder, prostate or gynaecological, pelvic soft tissue sarcomas	Glutamine	36	65.4
Kozjek, 2011 [23]	Rectal cancer	Glutamine	33	62.3
Kozelsky, 2003 [24]	Gynaecological, rectal or prostate	Glutamine	129	66.4

**PUFAs**				
Aredes, 2019 [36]	Cervical	EPA and DHA	42	44.5
Faramarzi, 2017 [37]	Rectal	CLA	26	60.2

**Polyphenols**				
Emami, 2014 [59]	Pprostate, uterus, cervical, bladder, rectal and colon)	Green tea	40	62.2
Ahmad, 2010 [34]	Prostate	Soy isoflavones	31	60–65*

### 3.2 Efficacy of dietary supplements in preventing diarrhoea

**Fig. 3:**
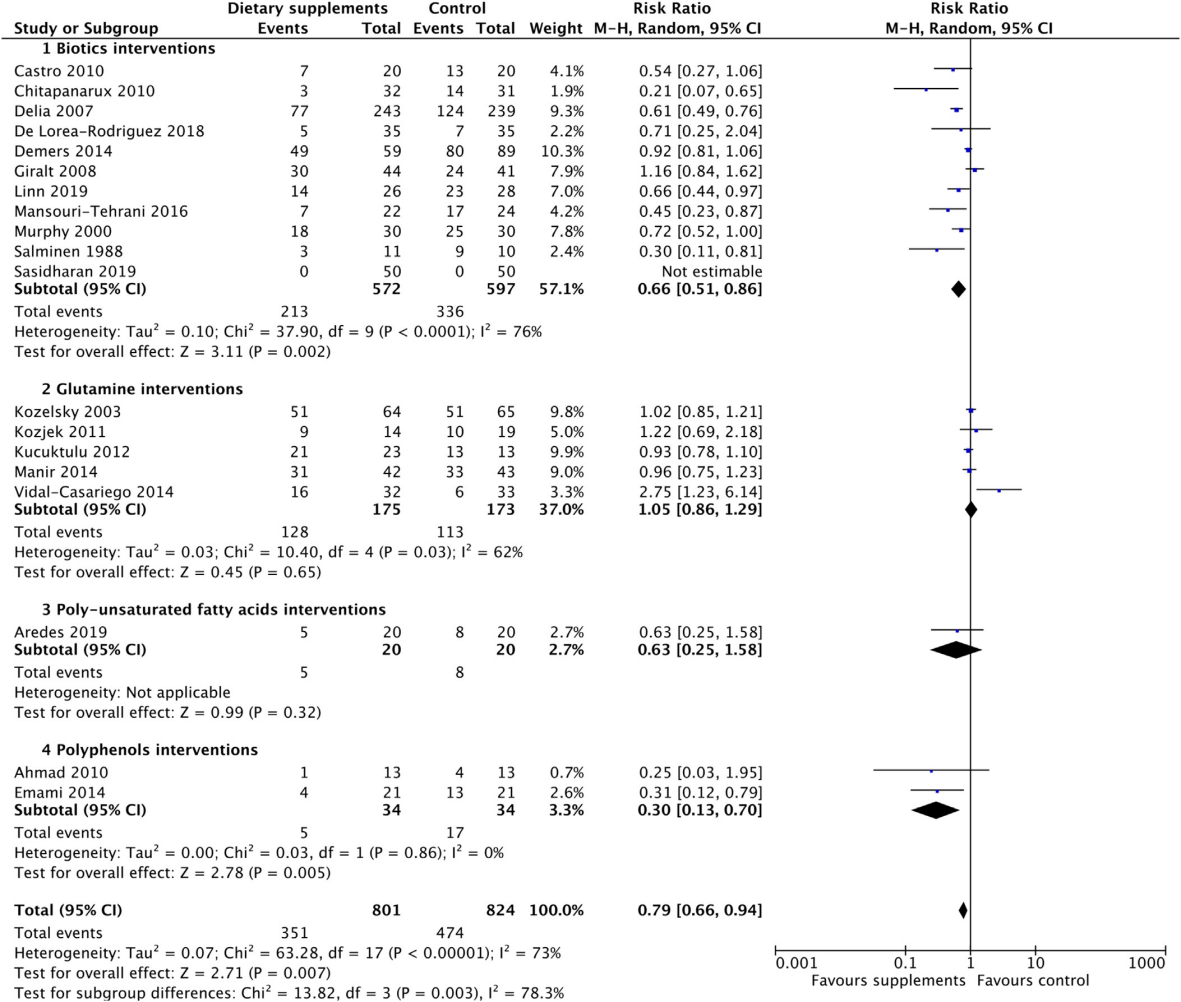
Forest plot of effects of biotic, glutamine, PUFA and polyphenol supplements on incidence of diarrhoea.

The meta-analysis comprising 1625 patients showed that dietary supplements reduced the risk of diarrhoea (Fig. 3). The overall pooled analysis showed significant heterogeneity amongst the studies (I^2^ = 73%; P < 0.001). Meta-analyses were carried out for biotic, glutamine, poly-unsaturated fatty acid and polyphenol interventions. Although the funnel plot for this meta-analysis (Figure S2) was largely symmetrical, the distributions of subgroup studies tended to be less symmetrical, implying moderate publication bias in the references included. There was no evidence that heterogeneity was due to mean age or sex of participants or sample size of the studies.

#### 3.2.1 Efficacy of biotics in preventing diarrhoea

Biotic interventions significantly reduced the risk of diarrhoea with a RR of 0.66 (95% CI: 0.51 to 0.86; P = 0.002) (Fig. 3). All studies, except Giralt *et al*[19], had a RR of less than 1, suggesting the protective role of biotics against diarrhoea. The heterogeneity, I^2^, among these studies was 76% (P < 0.001), so further analysis of the subclasses of probiotics and synbiotics was performed (Figure S3). The risk ratios were 0.45 (95% CI: 0.28 to 0.73) for synbiotics and 0.71 (95% CI: 0.52 to 0.99) for probiotics. Subgroup analysis was conducted by use of brachytherapy and chemotherapy (Figure S4 and S5). Patients not receiving brachytherapy (RR = 0.63; 95% CI: 0.54 to 0.73) or not receiving chemotherapy (RR = 0.62; 95% CI: 0.52 to 0.74) benefited from probiotics and synbiotics. With a smaller effect size, there was still a trend for those receiving brachytherapy (RR = 0.69; 95% CI: 0.41 to 1.15) or chemotherapy (RR = 0.72; 95% CI: 0.51 to 1.03).

#### 3.2.2 Efficacy of glutamine in preventing diarrhoea

Glutamine interventions were not associated with risk of diarrhoea with a RR of 1.05 (95% CI = 0.86 to 1.29; P = 0.65, Fig. 3). We found that four studies had consistent results of RR which were close to 1, but only Vidal-Cassariego *et al* reported a high RR of 2.75. There was high heterogeneity among studies (I^2^ = 62%, P = 0.03) [20].

#### 3.2.3 Efficacy of polyphenol in preventing diarrhoea

Two studies compared polyphenols and placebo among 64 patients (Fig. 3). Both showed that the intervention was associated with lower incidence of diarrhoea. The overall RR was 0.30 (95% CI = 0.13 to 0.70, P = 0.005). There was no evidence of heterogeneity between these two studies (I^2^ = 0%, P = 0.86).

### 3.3 Efficacy of dietary supplements in preventing moderate to severe diarrhoea

**Fig. 4:**
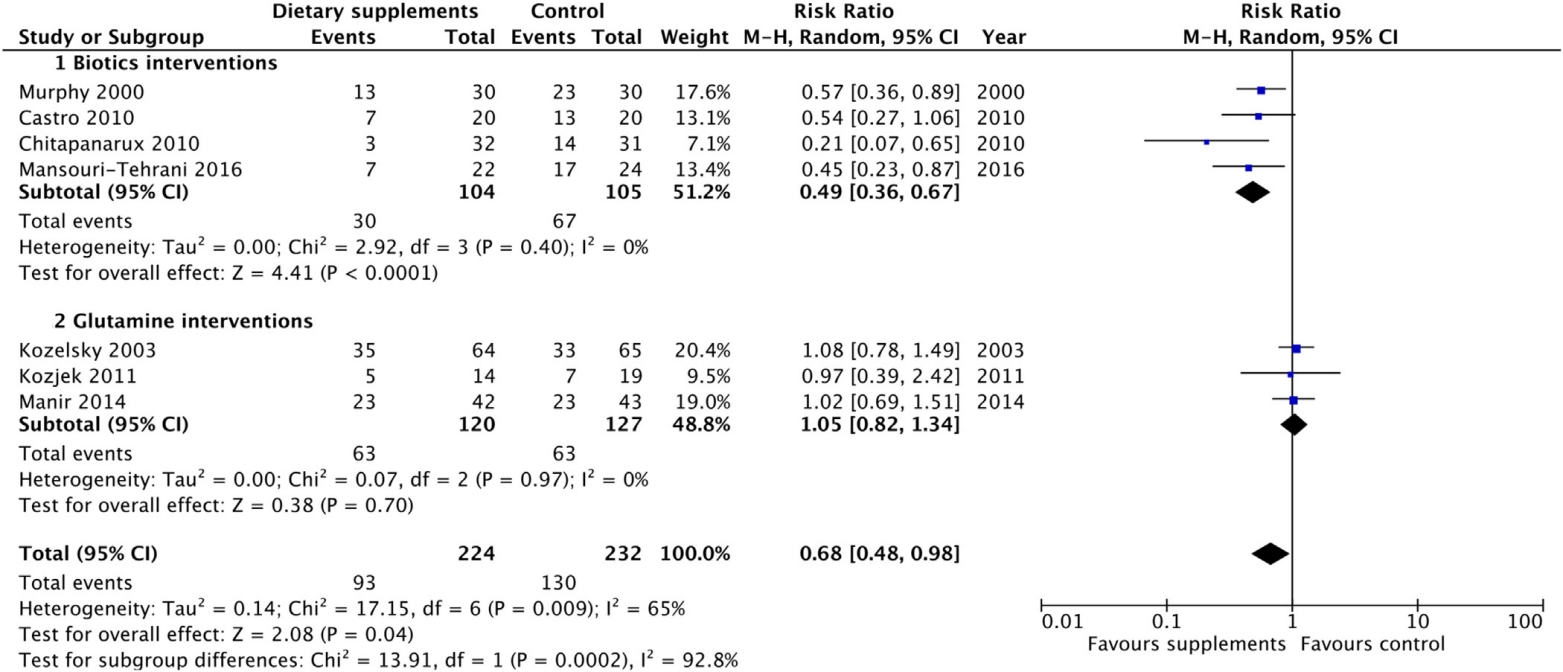
Forest plot of effect of dietary supplements on incidence of moderate to severe diarrhoea.

Efficacy of dietary supplements was assessed against moderate to severe diarrhoea, with this incidence defined as the incidence of grade 2 or higher diarrhoea, based on Common Terminology Criteria for Adverse Events (CTCAE) (older version: Common Toxicity Criteria; CTC)[21], except Murphy *et al* using the Murphy Diarrhoea Scale (MDS)[22] and Kozjek *et al* using their own criteria[23] (Fig. 4). The meta-analysis suggested that the association was mainly driven by biotic interventions for which the RR was 0.49 (95% CI: 0.36 to 0.67; P < 0.001), but not glutamine (RR = 1.05; 95% CI: 0.82 to 1.34; P = 0.70).

### 3.4 Efficacy of dietary supplements in preventing the use of anti-diarrhoeal medication

**Fig. 5:**
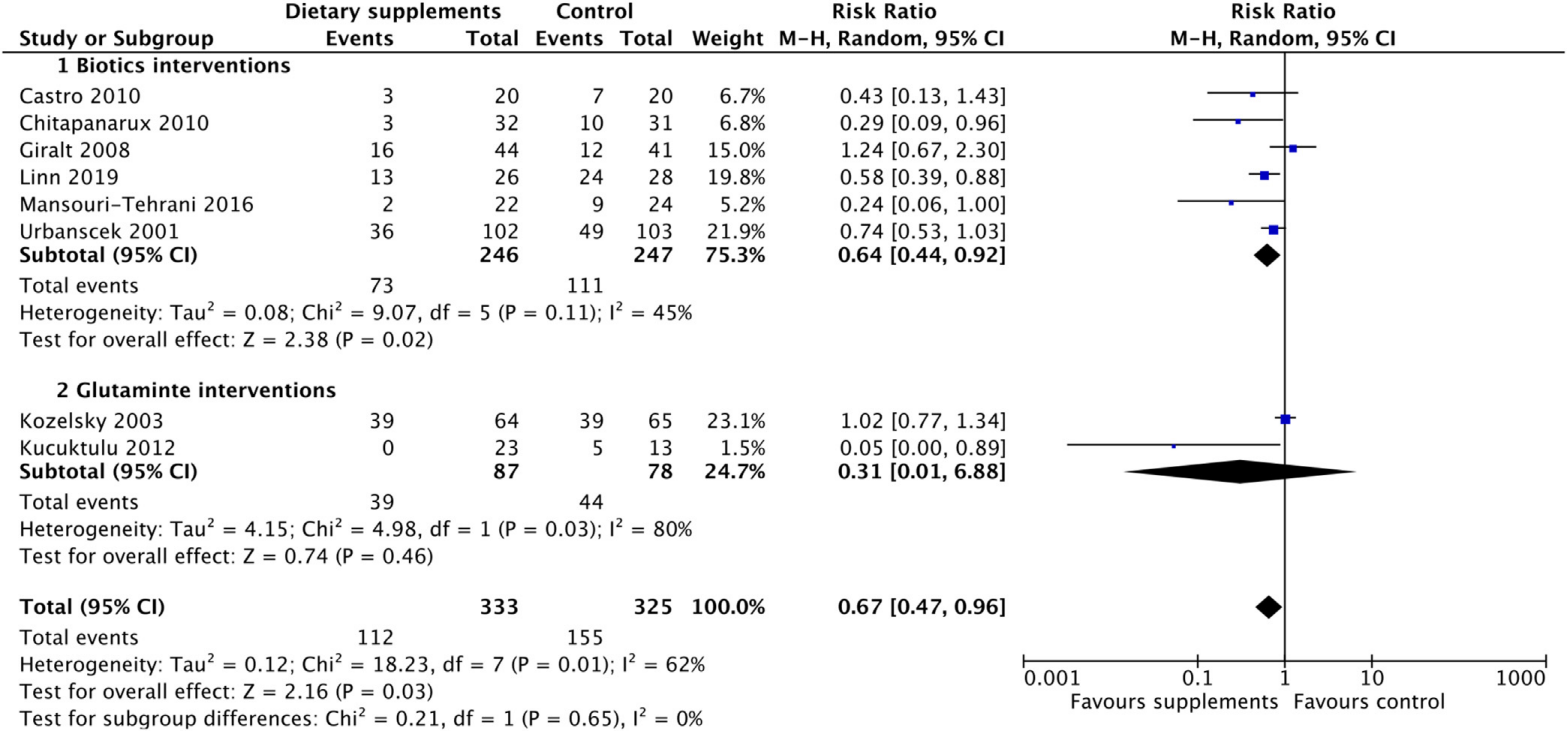
Forest plot of effect of dietary supplements on incidence of anti-diarrhoeal medication use.

Anti-diarrhoeal medication, such as loperamide, is often employed for patients who experience diarrhoea during or after radiotherapy. Therefore, we measured the effect of dietary supplements against the incidence of anti-diarrhoeal medication use (Fig. 5), and found that biotic interventions were associated with lower risk of anti-diarrhoeal medication use in patients (RR = 0.64; 95% CI: 0.44 to 0.92; P = 0.02) and there was intermediate heterogeneity among studies (I^2^ = 45%; P = 0.11).

### 3.5 Effects of dietary supplements on nausea, vomiting, flatulence/bloating, bowel movement frequency, tenesmus and blood in bowel movement

As shown in Table S4, dietary supplements tended to decrease the risk of nausea (RR = 0.74; 95% CI: 0.36 to 1.50; P = 0.40) and the mean number of bowel movements per day (mean difference = −3.88; 95% CI: −10.29 to 2.52; P = 0.23). The results also showed that the interventions had no effect on vomiting and flatulence/bloating with relative risks of 0.99 (95% CI: 0.79 to 1.25, P = 0.95) and 1.12 (95% CI: 0.59 to 2.12; P = 0.72) respectively. Only Kozelsky *et al* studied the outcomes of tenesmus and blood in bowel movements and found that glutamine had no effect on these symptoms [24].

## 4 Discussion

This review showed that dietary supplements are effective in reducing the risk of diarrhoea, experiencing moderate to severe diarrhoea and anti-diarrhoeal medication use, in the acute setting following radiotherapy. Subgroup analysis showed that biotic supplements and polyphenols were effective in reducing the risk of these outcomes, but glutamine was ineffective. Among the subclasses of biotic interventions, both probiotic and synbiotic supplements were shown to be effective in reducing the risk of diarrhoea, particularly among patients not receiving brachytherapy (p < 0.001) or chemotherapy (p < 0.001; Figure S4A and S5A). Although the fibre types of prebiotics included in this systematic review were heterogenous, the bacterial genera of probiotics and synbiotics were homogenous, as they contained *Lactobacillus* and *Bifidobacteria* only.

Several meta-analyses have been conducted regarding probiotic and synbiotic supplements over the last decade, but neither the category of prebiotics nor subgroups of patients receiving brachytherapy or chemotherapy have yet been studied. The previous meta-analyses investigating the effects of biotic supplements on acute symptoms of gastrointestinal toxicity are listed in Table S5 [25], [26], [27], [28], [29]. A Cochrane systematic review has investigated the efficacy of interventions, including radiotherapy techniques and pharmacological and non-pharmacological interventions, including dietary interventions, probiotics, glutamine, counselling, and protein supplements, on acute and late adverse gastrointestinal effects of pelvic radiotherapy for primary pelvic cancers [3]. In contrast, we took a more focused approach, and within our parameters showed that probiotics and synbiotics were the most beneficial interventions. Our search to June 2020 included three more recent studies of biotic supplements (224 patients) and one study focusing on PUFA supplements (40 patients), compared to the Cochrane study, whose search only extended to November 2017. Currently, there are no published meta-analyses that investigate the effect of PUFA or polyphenol supplements on acute symptoms of gastrointestinal toxicity, and two included studies of polyphenols suggested that they are beneficial in preventing diarrhoea.

Studies have shown that the risk factors for radiation enteritis include older age [4], dose of radiation used [30], combining internal (brachytherapy) and external RT [31] and the concomitant use of chemotherapy [32]. Figure S1 show that the effects of interventions on incidence of diarrhoea did not vary by mean age (A) or RT dose (D). Seventeen out of 23 studies used an RT dose of approximately 50 Gy, the exceptions being Delia *et* al [33], Murphy *et al* [22], Ahmad *et al* [34] which used differing higher doses, and 3 studies that did not specify the dosage, including Castro *et al* [35], Aredes *et al* [36] and Faramarzi *et al* [37], but details of techniques were limited. Few studies documented the use of more modern radiotherapy techniques, including intensity modulated radiotherapy. In the Cochrane systematic review [3], such modern techniques, including IMRT and 3D-conformal radiotherapy resulted in lower acute gastrointestinal toxicity than older techniques, and there was uncertain evidence for superiority of IMRT over 3D-conformal. However, high-dose IMRT can still perturb the gut microbiota by reducing its diversity [38]. Therefore, we hypothesise that, as probiotics and synbiotics can positively augment favourable gut microbiota colonization [39], their use will still have an impact in the modern radiotherapy era.

To measure the incidence of diarrhoea, 13 studies used the scale of CTC or CTCAE. It is noted that CTCAE assesses ‘diarrhoea’ by an increase in frequency and/or loose or watery bowel movements [40]. The other studies used either Bristol stool form scale (BSFS), WHO toxicity grading, Radiation Therapy Oncology Group (RTOG) toxicity scale, Murphy Diarrhea Scale (MDS), adapted NCI questionnaire, European Organization for Research and Treatment of Cancer Quality of Life Questionnaire version 3.0 (EORTC QLQ-C30) or non-specified Quality of life (QOL) questionnaire. These have similar definitions of diarrhoea, which enabled us to combine these trials (Table S2). The heterogeneity found in the quality assessment (Table S6), may reduce the evidence certainty of this study. Therefore, in future, methodologically well‐designed, large-scale trials are needed to strengthen the evidence for the benefits of dietary supplements.

Preclinical studies have shown that prebiotics can enhance the efficacy of chemotherapy and radiotherapy [41], [42], in terms of tumour control. As most modern neoadjuvant/radical pelvic radiotherapy regimens (except prostate cancer) include chemotherapy, and with the current interest in combined brachy-EBRT dose-escalation in prostate cancer, the future clinical applicability of biotics in clinical practice should be rigorously evaluated in terms of both tumour control and sparing of normal tissue toxicities in these modern settings.

The underlying protective effects of dietary supplements against GI toxicities may be mediated as shown in Figure S6. A direct effect on the intestinal immune environment following intake of specific dietary agents may lead to anti-inflammatory changes that alleviate gastrointestinal toxicity. There may also be an indirect effect, whereby the above immunomodulatory actions are developed in response to changes in the gut microbiota and their metabolites, particularly SCFAs. A systematic review conducted by Tonneau *et al* emphasised the importance of probiotics for gastro-intestinal toxicities as radiotherapy can cause perturbation of gut microbiota [43].

Limitations of our study include the different methods of morbidity assessment used in different studies (see above), the difficulty in disentangling radiotherapy side effects from the occurrence of independent gastrointestinal symptoms, the lack of detailed radiotherapy dose parameters available, and the use of different pelvic malignancies requiring different target volumes within the pelvis, which may influence the severity of gastrointestinal side effects.

This review aimed to investigate the effect of dietary supplements on acute and late symptoms, but no studies were available reporting on late side effects. Chronic symptoms of gastrointestinal toxicity typically emerge a few months to years following irradiation and occur in most of the intestinal compartments [44]. Evidence from clinical studies suggests that acute and chronic effects are linked, with the risk of developing late effects greater in patients that have developed acute effects (consequential late effects) [45], [46].

## 5 Conclusion

In conclusion, findings from our systematic review and meta-analysis suggest that biotic supplements, specifically probiotics and synbiotics, are effective in reducing the risk and severity of acute symptoms of gastrointestinal toxicity caused by pelvic radiotherapy. Our study highlights the need for large multi-centre clinical trials of biotic interventions in patients undergoing radiation and chemoradiation treatments, using modern radiotherapy techniques, with detailed dosimetry of external beam radiotherapy and brachytherapy and appropriate acute and late outcome measures.

## Funding

This work was supported by Cancer Research UK Programme grant [C5255/A23755]. Chee Kin Then’s DPhil is funded by the Clarendon Fund, Balliol College and CRUK. The funding body had no role in the study design, collection, analysis, interpretation of data or in writing the manuscript.

## Declaration of Competing Interest

The authors declare that they have no known competing financial interests or personal relationships that could have appeared to influence the work reported in this paper.
